# Regional integration and public healthcare environment: Evidence from China

**DOI:** 10.3389/fpubh.2022.1013053

**Published:** 2023-01-04

**Authors:** Chenglin Tu, Yonghui Zeng, Hongyu Long, Chenyang Yu, Yuanfang Tan, Yu Zhou, Chuanxiang Zang

**Affiliations:** ^1^School of Economics and Statistics, Guangzhou University, Guangzhou, China; ^2^Academy of Guangzhou Development, Guangzhou University, Guangzhou, China; ^3^School of Management, Guangzhou University, Guangzhou, China; ^4^Department of Social Work, School of Public Administration, South China Agricultural University, Guangzhou, China; ^5^Nanyang Technopreneurship Centre, Nanyang Technological University, Singapore, Singapore

**Keywords:** regional integration, public healthcare environment, hierarchical linear model, spatial core-peripheral cities, heterogeneity analysis

## Abstract

**Introduction:**

Existing studies have focused on the impact of economic development and urban expansion on public healthcare environment but has ignored the importance of regional integration. Regional integration reflects the spatial distribution of the labor force, which significantly affects healthcare workforce and healthcare infrastructure development.

**Methods:**

Based on panel nested data for 137 cities in 16 major city clusters in China from 2001 to 2019, this paper assesses the impact of regional integration on the public healthcare environment through a hierarchical linear model (HLM).

**Results:**

Our findings indicate that a 1% increase in regional integration leads to a 6.6 and 1.9% improvement in healthcare workforce and healthcare infrastructure. The results of the mechanism analysis indicate that regional integration affects the public healthcare environment through improving transportation infrastructure and industrial upgrading. In addition, regional integration has a stronger effect on cities with lower levels of economic development and healthcare environments. Finally, the endogeneity test based on the difference-in-difference (DID) model and the robustness test based on high-dimensional fixed effects model conduct the consistent conclusions.

**Discussion:**

Policies to improve the public healthcare environment through promoting regional integration are proposed. Government should develop a more comprehensive regional cooperation plan to improve the public healthcare environment. Also, financial spending on improving the healthcare environment in peripheral cities should be increased. In addition, regional integration policy development needs to consider differences across regions.

## 1. Introduction

The COVID-19 pandemic and increased environmental pollution are a constant threat to public health (1, 2). Upgrading the public healthcare environment is the most powerful measure to address the challenge (3, 4). The impact of various factors on enhancing the public healthcare environment has been explored in the literature. Swift pointed out that the level of regional economic development is an important factor affecting the healthcare environment (5). Holecki et al. pointed out that population size and structure are also thought to significantly impact the healthcare environment (6). In addition, some scholars also proposed that foreign investment, government intervention, information and communication technology development and government investment in education are all essential for the improvement of healthcare environment (7–15). Countries have started to improve the healthcare environment through the above channels, but there is still needed to find new driving factors to further enhance the healthcare environment.

With the global economic slowdown, more and more scholars are focusing on the evolution of regional integration rather than aggregate growth. The process of regional integration will unlock potential demand and effectively reduce product transaction costs, which will promote social and economic development (16). As a representative country in the shift from a planned economy to a market economy, China's reform and opening up in 1978 effectively facilitated the process of regional integration. Regional integration in China refers to the process of breaking down barriers to factor movement between different regions (17). According to the study of Duranton and Puga and Kang et al., the regional integration in China can be reflected by the division of labor pattern in which core cities dominate R&D while peripheral cities dominate processing and manufacturing (18, 19). A higher degree of the above division of labor indicates lower barriers to factor mobility within the region, i.e., a higher degree of regional integration. With regional integration, labor, capital and technology can move among different cities in the region at a lower cost. The above factors will flow to the region with the most comparative advantage through the price mechanism, thus promoting regional socio-economic development. In addition, due to the extensive regional economic development imbalance in China, the degree of regional integration is significantly higher in coastal areas than inland areas (19). Therefore, this paper will further explore the differences in the impact of regional integration in different regions.

Regional integration will not only affect economic development, but its impact on the healthcare environment cannot be ignored. The development of regional integration can effectively utilize the comparative advantages of different cities and fully release the economic development potential (20). In addition, regional integration is also the optimization of labor spatial distribution through the market mechanism (21). Economic development and population migration are important factors affecting the public healthcare environment (9, 22). On the one hand, the increase of economic development level will enhance the investment of financial and private capital in public health, thus improving the public medical health environment (7, 22). On the other hand, population migration will lead to changes in healthcare demand, which in turn will affect the public healthcare environment (23, 24). However, regional integration has not received attention as a potentially important factor affecting the public healthcare environment. To fill this research gap, this paper collects panel data of 137 cities in 16 major cities in China from 2001 to 2019 to examine the impact of regional integration and public healthcare environment through a hierarchical linear model (HLM). Meanwhile, this paper further explores the differences between the impact of regional integration on core cities and peripheral cities. Finally, this paper enhances the robustness of this study through heterogeneity analysis, endogeneity test and robustness test.

The contribution of this paper is mainly reflected in the following three points. First, this paper explores the improvement of public healthcare environment from the perspective of regional integration. Existing studies have focused on the impact of factors such as medical technology and economic development on the public healthcare environment. To the best of our knowledge, there are no studies linking regional integration to the public healthcare environment. This paper points out that regional integration promotes the comparative advantages of core cities and peripheral cities, thus improving the public healthcare environment through economic development and labor force division. Second, this paper applies HLM to estimate the impact of regional integration on public healthcare environment. In this paper, regional integration is a cluster-level indicator and public healthcare environment is a city-level indicator, i.e., this paper uses nested data of city cluster and city. Studies on the public healthcare environment have been based on OLS estimation at the city or provincial level. In contrast, for nested data, OLS estimation ignores the effect of differences in stratified data. HLM can reduce this bias to a certain extent. Finally, this paper uses the Yangtze River Delta Expansion as a quasi-natural experiment for endogeneity testing to ensure the robustness of the results.^1^ Studies have used the functional division of labor index to measure regional integration. However, the public healthcare environment also affects labor migration to some extent, which in turn affects regional integration. The resulting endogeneity bias reduces the reliability of the estimation results. To reduce the interference of endogeneity on the findings, this paper uses the exogenous policy shock of the Yangtze River Delta Expansion as the dependent variable and assesses its impact on the public healthcare environment based on difference-in-difference (DID) model.

The reminder of this study is organized as follows: Section 2 provides the research hypothesis, Section 3 provides the methods and data, Section 4 provides the results, and Section 5 provides the conclusions and recommendations.

## 2. Research hypothesis

The studies of Duranton and Puga and Kang et al. pointed out that the city cluster presents a specialization division of labor in which manufacturing concentrated in peripheral cities and R&D concentrated in core cities, i.e., regional integration (18, 19). The evolution of this division of labor pattern can have a significant impact on the public healthcare environment in cities across the region. As rapid economic growth can lead to the enhancement of social aspects, on the one hand, there will be a significant improvement in transportation infrastructure (25). Regional integration requires regions joining the alliance to build faster information channels and easier ways to exchange resources to meet the growing demand for economic development (26). In turn, the construction of a healthcare environment requires a more efficient flow of healthcare resources. For example, a good healthcare environment will ensure emergency vehicles reach their destination in a short enough time. This means that an upgraded transportation infrastructure can contribute significantly to optimizing the response time of healthcare resources (27, 28). Therefore, regional integration can enhance the healthcare environment through improved transportation infrastructure. On the other hand, regional integration can lead to significant industrial upgrading (29). As regional integration allows for a freer and more rational allocation of resources, the industrial structure will change along with the type of companies in the region. The tertiary service industry can have a greater effect because of the higher demand for resource exchange and cooperative innovation (30). Therefore, regional integration can significantly increase the proportion of the tertiary sector in the structure. Compared with the primary and secondary industries, the construction of the healthcare environment is more closely related to the tertiary industry. The deployment of related medical facilities and the conduct of medical activities all require cooperation with the service industry. It means that the increase in the proportion of service industries also promotes the development of healthcare environment by supporting from subsidiary industries. Therefore, regional integration can enhance the healthcare environment by promoting the upgrading of industrial structure. Based on the above analysis, this paper proposes hypothesis 1 and hypothesis 2:

H1: Regional integration has positive impact on the urban public healthcare environment.
H2: Regional integration improves the healthcare environment through improving transportation infrastructure and industrial upgrading.

It is important to note that there may be differences in the impact of regional integration on the public healthcare environment in core and peripheral cities in the region. While all cities in the region can benefit from the increased level of regional integration, the core cities have a stronger effect in enhancing the public healthcare environment. First, increased regional integration will further strengthen the concentration of high-end productive services in the core cities (18, 19). The resulting concentration of talent raises the demand for high-quality healthcare in core cities. This will significantly promote the development of public healthcare environment in core cities. Second, regional integration will promote the transfer of high-end manufacturing industries from the core cities to the peripheral cities (31). The upgrading of manufacturing industries in peripheral cities can also attract talents and improve the public healthcare environment. However, compared with the core cities, the talent gathering effect brought by the upgrading of manufacturing industry in peripheral cities is significantly lower than that of production service industry (32, 33). In the process of regional integration, the public healthcare environment improvement is stronger in the core cities where human capital is the main production factor. Therefore, the following hypothesis 3 is proposed in this paper:

H3: Regional integration has a higher effect on public healthcare environment in core cities than in peripheral cities.

## 3. Methods and data

### 3.1. Data

This paper chooses 16 city clusters in China as the research object, including a total of 137 prefecture-level cities. All the cities in these city clusters are shown in Table A1 and Figure A1 in Supplementary material. Although there are 24 city clusters in China, some of them were established earlier and have serious data deficiencies, which will reduce the reliability of the estimation results (19). Therefore, this paper excluded eight city clusters (19). The sample data cover the period from 2001 to 2019. Data are collected from Chinese City Statistics Database (CCSD) in Chinese Research Data Services (CNRDS) Platform (https://www.cnrds.com/Home/Index#/) and China Statistical Yearbook (2002–2020).

### 3.2. Empirical model

This paper focuses on the impact of regional integration on the public healthcare environment. For nested data containing both city cluster-level and city-level data, HLM is more suitable for parameter estimation than OLS or panel regression, which ignore the hierarchical structure of nested data. One of the consequences is that the standard errors of the parameters are underestimated, leading to an overestimation of the significance of the coefficients (34). According to the study of Chen and Jou (35), this paper constructs the following three models:

The first model is null model (NM), which is employed to analyze the necessity of using HLM. According to Raudenbush and Bryk (36), NM is specified:


(1) $$\begin{array}{l} \text{Level }1:\ln\;phe_{i,j,t}=\varphi_{0j}+\varepsilon_{ijt} \end{array}$$

(2) $$\begin{array}{l} \text{Level }2:\;\varphi_{0j}=\tau_{00}+\mu_{0j} \end{array}$$

(3) $$\begin{array}{l} \text{Mixed model}:\ln\;phe_{i,j,t}=\tau_{00}+\mu_{0j}+\varepsilon_{ijt} \end{array}$$

where *lnphe*_*i,j,t*_ denotes the logarithmic value of public healthcare environment of city *i* in cluster *j* at year *t*. φ_0*j*_ denotes the random effect between city clusters, τ_00_denotes the average effect of all the city clusters, Level 1 denotes the city level, and level 2 denotes the city cluster level. The intra-cluster correlation coefficient (ICC) can be calculated by the following Eq. (4), which reflects the contribution rate of intra-cluster correlation coefficient differences to differences in public healthcare environment.


(4) $$\begin{array}{l} ICC=\frac{{\sigma_{2}}^{2}}{{\sigma_{2}}^{2}+{\sigma_{1}}^{2}} \end{array}$$

where ${\sigma_{1}}^{2}$ is the intra-cluster variance at the level 1,${\sigma_{2}}^{2}$ is the inter-cluster variance at the level 2. ICC is used to justify the application of HLM (35). Lower value of ICC means a smaller difference in public healthcare environment between different city clusters. In this case OLS is more appropriate than HLM. However, when the value of ICC is high, HLM is more appropriate than OLS. The study of Ozkaya et al. shows that HLM can be used when ICC is higher than 0.1 (34).

Random-interpret and random-slope model (RIRSM) considers city-level control variables and cluster-level independent variable based on NM, which is specified:


(5) $$\begin{array}{l} \text{Level }1:\ln\;phe_{i,j,t}=\varphi_{0j}+\varphi_{1j}Con_{i,j,t}+\varepsilon_{ijt} \end{array}$$

(6) $$\begin{array}{l} \text{Level }2:\begin{array}{c} \;\varphi_{0j}=\tau_{00}+\tau_{01}ri_{j,t}+\mu_{0j}\\ \varphi_{1j}=\tau_{10}+\mu_{1j} \end{array} \end{array}$$

(7) $$\begin{array}{l} \text{Mixed model}:\begin{array}{c} \;\ln\;phe_{i,j,t}=[\tau_{00}+\tau_{01}ri_{j,t}+(\tau_{10}\\ +\mu_{1j})\times X_{i,j,t}]\\ +\left[\varepsilon_{ijt}+\mu_{0j}\right] \end{array} \end{array}$$

where *Con*_*i,j,t*_ denotes city-level factors including, *ri*_*j,t*_ denotes the degree of regional integration of city cluster, φ_1*j*_ denotes the slopes of city-level factors, τ_01_ denotes the final coefficient of *ri*_*j,t*_ of city cluster.

This paper further constructs random-interpret and random-slope (with interaction) model (RIRSIM) to explore the impact of regional integration on the slope of each city-level factor, which is specified:


(8) $$\begin{array}{l} \text{Level }1:\ln\;phe_{i,j,t}=\varphi_{0j}+\varphi_{1j}Con_{i,j,t}+\varepsilon_{ijt} \end{array}$$

(9) $$\begin{array}{l} \text{Level }2:\begin{array}{c} \;\varphi_{0j}=\tau_{00}+\tau_{01}ri_{j,t}+\mu_{0j}\\ \varphi_{1j}=\tau_{10}+\tau_{11}ri_{j,t}+\mu_{1j} \end{array} \end{array}$$

(10) $$\begin{array}{l} \text{Mixed model}:\begin{array}{c} \;\ln\;phe_{i,j,t}=[\tau_{00}+\tau_{01}ri_{j,t}+\left(\tau_{10}+\mu_{1j}\right)\\ \times Con_{i,j,t}+\tau_{11}ri_{j,t}\times Con_{i,j,t}]\\ +\left[\varepsilon_{ijt}+\mu_{0j}\right] \end{array} \end{array}$$

where τ_11_ denotes the impact of regional integration of city cluster on the slope of city-level factors. This paper further analyzes the impact of regional integration on the slopes of various city-level factors through this model.

### 3.3. Variables

#### 3.3.1. Dependent variable

The dependent variable in this paper is the public healthcare environment. Based on the study of Song et al. (9), this paper measures the public healthcare environment in terms of the healthcare workforce and the healthcare infrastructure. Specifically, this paper measures healthcare workforce through the logarithm of the total number of doctors, and higher values of this indicator represent a better public healthcare environment. In addition, the logarithm of the number of hospitals beds is used to measure the healthcare infrastructure, and a higher value of this indicator indicates a better public healthcare environment. The number of hospitals or the number of medical facilities is more reflective of the urban healthcare infrastructure, but the above data are not reported. In contrast, the number of hospital beds in a city is highly correlated with the number of hospitals and the number of medical facilities. This means that the number of hospital beds also effectively reflects the level of urban healthcare infrastructure. Since the data of city-level hospital beds has more comprehensive and continuous publication, this paper selected this indicator as the proxy variable for healthcare infrastructure.

#### 3.3.2. Independent variable

Regional integration is the independent variables in this paper. We employ the function urban specialization index proposed by Duranton and Puga and Kang et al. to measure the degree of regional integration of the city cluster (18, 19), which is specified:


(11) $$\begin{array}{l} ri_{j,t}=\frac{\overset{nc}{\underset{i=1}{\sum }}lsc_{i,j,t}/\overset{nc}{\underset{k=1}{\sum }}lmc_{i,j,t}}{\overset{np}{\underset{i=1}{\sum }}lsp_{i,j,t}/\overset{np}{\underset{i=1}{\sum }}lmp_{i,j,t}} \end{array}$$

where *i* = 1, 2……*nc* denotes core cities in urban agglomeration *j, i* = 1, 2……*np* denotes peripheral cities in city cluster *j, lsc*_*i,j,t*_ denotes the number of employees in the producer service industry of core city *i* in year *t, lmc*_*i,j,t*_ denotes the number of employees in the manufacturing industry of core city, *lsp*_*i,j,t*_ denotes the number of employees in the producer service industry of peripheral city, *lmp*_*i,j,t*_ denotes the number of employees in the manufacturing industry of peripheral city. *ri*_*j,t*_ denotes the degree of regional integration, and higher level of *ri*_*j,t*_ means higher degree of regional integration.

#### 3.3.3. Mediating variables

According to the research hypothesis in Section 2 of this paper, regional integration improves the healthcare environment by influencing transportation infrastructure and industrial upgrading. Therefore, transportation infrastructure level (ln*tran*) and industrial structure (*is*) are selected as mediating variables to test the mechanism of regional integration affecting healthcare environment in this paper. The industrial structure is measured by the proportion of tertiary industry output to GDP, and transportation infrastructure level is measured by the logarithm of road area per capita (37).

#### 3.3.4. Control variables

To assess the impact of regional integration on the public healthcare environment more reliably, this paper controls for a series influencing factors of public healthcare environment, including information and communication technology (ln*ict*), level of foreign investment (*fdi*), government investment in education (*gie*), population (ln*pop*), government intervention (*gov*) and economic development level (ln*rgdp*) (7–11, 38, 39). The measurement and descriptive statistics for all variables are shown in Table 1.

**Table 1:** Descriptive statistics.

**Variables**		**Variable description**	***N***	**Mean**	**Min**	**Max**	**Std**
Dependent variable	ln*phe1*	Logarithmic value of number of doctors	2,558	8.932	6.617	11.659	0.788
	ln*phe2*	Logarithmic value of number of hospital beds	2,558	9.575	7.187	12.086	0.769
Independent variable	*ri*	Degree of regional integration	2,558	1.297	0.617	0.464	3.778
Mediating variable	ln*rgdp*	Logarithmic value of GDP per capita	2,558	10.321	7.854	13.056	0.880
	*is*	Percentage of total output value of tertiary industry	2,558	0.504	0.154	0.844	0.117
Control variable	ln*ict*	Logarithm of the number of Internet users	2,558	5.978	4.240	8.134	0.636
	ln*pop*	Logarithmic value of population	2,558	12.879	5.468	17.762	1.374
	*fdi*	Foreign direct investment/GDP	2,558	0.004	0.000	0.032	0.004
	*gie*	Education expenditure/ government expenditure	2,558	0.179	0.044	0.494	0.043
	*gov*	Government expenditure/GDP	2,558	0.134	0.028	0.675	0.061
	ln*rgdp*	Logarithmic value of GDP per capita	2,558	10.321	7.854	13.056	0.880

## 4. Results

### 4.1. Impact of regional integration on public healthcare environment

Table 2 reports the estimated results of the impact of regional integration on the public healthcare environment based on HLM. ln*phe1* and ln*phe2* denote the level of healthcare workforce and healthcare infrastructure, respectively. Columns (1) and (4) report the regression results for NM, columns (2) and (5) for RIRSM, and columns (3) and (6) for RIRSIM.

**Table 2:** Impact of regional integration on public healthcare environment.

	**ln*phe1***			**ln*phe2***		
	**NM**	**RIRSM**	**RIRSIM**	**NM**	**RIRSM**	**RIRSIM**
	**(1)**	**(2)**	**(3)**	**(4)**	**(5)**	**(6)**
**Fixed effect**						
• *ri*		0.066^***^	−0.809^***^		0.019^**^	−0.313
		(0.011)	(0.246)		(0.009)	(0.207)
• ln*ict*		0.128^***^	0.071^**^		0.132^***^	0.095^***^
		(0.011)	(0.029)		(0.009)	(0.025)
• *gie*		−0.514^***^	−1.028^**^		−1.256^***^	−1.345^***^
		(0.156)	(0.423)		(0.126)	(0.356)
• *fdi*		−13.178^***^	−15.739^***^		−4.710^***^	−16.472^***^
		(1.858)	(4.985)		(1.504)	(4.190)
• ln*pop*		0.875^***^	0.906^***^		0.852^***^	0.884^***^
		(0.014)	(0.035)		(0.012)	(0.030)
• *gov*		0.958^***^	−1.455^***^		0.080	−0.848^***^
		(0.144)	(0.291)		(0.089)	(0.244)
• ln*rgdp*		0.383^***^	0.168^***^		0.218^***^	0.237^***^
		(0.020)	(0.040)		(0.013)	(0.033)
**Random effect**						
• *ri* × ln*ict*			0.025			−0.009
			(0.030)			(0.025)
• *ri* × *gie*			0.053^**^			0.031
			(0.022)			(0.019)
• *ri* × *fdi*			−0.029			0.136
			(0.326)			(0.274)
• *ri* × ln*pop*			12.319^***^			9.879^***^
			(3.690)			(3.101)
• *ri* × *gov*			−0.045^*^			−0.028
			(0.025)			(0.021)
• *ri* × ln*rgdp*			0.729^***^			0.773^***^
			(0.235)			(0.198)
• Constant	8.892^***^	−1.403^***^	1.291^***^	9.517^***^	0.744^***^	1.044^***^
	(0.062)	(0.183)	(0.320)	(0.059)	(0.102)	(0.269)
• ${\sigma_{\mu 0}}^{2}$	0.720^***^	0.325^***^	0.268^***^	0.693^***^	0.261^***^	0.263^***^
	(0.044)	(0.023)	(0.018)	(0.042)	(0.018)	(0.018)
• σ^2^	0.315^***^	0.176^***^	0.189^***^	0.330^***^	0.138^***^	0.137^***^
	(0.004)	(0.003)	(0.003)	(0.005)	(0.002)	(0.002)
• City FE	Y	Y	Y	Y	Y	Y
• Year FE	Y	Y	Y	Y	Y	Y
• Observations	2,558	2,558	2,558	2,558	2,558	2,558
• Likelihood ratio	4,063.66	1,777.90	1,789.89	3,724.25	2,453.23	2,451.94

Standard errors are reported in in parentheses.

***, **, and * denote p < 0.01, p < 0.05 and p < 0.1.

$\sigma_{\mu 0}^{2}$ and σ^2^ denote intra-cluster variance and the inter-cluster variance. Likelihood ratio denotes the test comparing HLM and linear model.

Based on the estimation results of NM, the ICC can be calculated to determine whether HLM should be used for estimation. According to the results in (1) and (4), the ICC is 0.3043 (0.315/ (0.315+0.720) = 0.3043) and 0.3226 (0.330/ (0.330+0.693) = 0.3226), respectively, indicating that 30.43 and 32.26% of the total difference in the level of healthcare workforce and healthcare infrastructure, respectively, is caused by urban clusters which is much higher than 10%. Therefore, HLM is more suitable than OLS for estimating the impact of regional integration on the public healthcare environment.

According to the estimation results in column (2), the regression coefficient of *ri* is 0.066 (*p* < 0.01), which indicates that regional integration significantly enhances the size of the medical workforce in the cities in the region. The regression coefficient of *ri* in column (5) is 0.019 (*p* < 0.01), indicating that regional integration significantly improves the level of medical infrastructure. Therefore, regional integration can significantly enhance the public healthcare environment in the region. This paper further explores the moderating effect of regional integration on other city-level influences. In the regression results in column (3), the regression coefficients of *ri* × *gie, ri* × ln*pop, ri* × *gov*, and *ri* × ln*rgdp* are 0.053 (*p* < 0.05), 12.319 (*p* < 0.01), −0.045 (*p* < 0.1) and 0.729 (*p* < 0.01), respectively. This suggests that increased regional integration significantly enhances the positive impact of education level, population and economic development level on the healthcare workforce. However, regional integration also amplifies the negative impact of government intervention on the healthcare workforce. In the regression results in column (6), the regression coefficients of *ri* × ln*pop* and ri × ln*rgdp* are 9.879 (*p* < 0.01) and 0.773 (*p* < 0.01), respectively. This indicates that the increase in regional integration significantly enhances the positive impact of population and economic development on healthcare infrastructure. In conclusion, we find that regional integration both directly improves the public healthcare environment of cities in the region and enhances the effect of population and economic development levels on the enhancement of the public healthcare environment.

### 4.2. Analysis of the mechanism of regional integration affecting healthcare environment

To test hypothesis 2, i.e., whether regional integration enhances the healthcare environment by improving transportation infrastructure and industrial upgrading, this paper conducted a mediating effects test. The estimated results are shown in Table 3.

**Table 3:** Analysis of the mechanism of regional integration affecting healthcare environment.

	**ln*tran***	**ln*phe1***	**ln*phe1***	***is***	**ln*phe2***	**ln*phe2***
	**(1)**	**(2)**	**(3)**	**(4)**	**(5)**	**(6)**
• *ri*	0.304^***^	0.094^***^	0.019^**^	0.040^***^	0.098^***^	0.021^**^
	(0.032)	(0.011)	(0.009)	(0.004)	(0.011)	(0.009)
• ln*tran*		0.029^***^	0.023^***^			
		(0.008)	(0.007)			
• *is*					0.359^***^	0.097^*^
					(0.061)	(0.051)
• ln*ict*		0.127^***^	0.132^***^		0.123^***^	0.130^***^
		(0.011)	(0.009)		(0.011)	(0.009)
• *gie*		−1.104^***^	−1.233^***^		−1.114^***^	−1.251^***^
		(0.151)	(0.126)		(0.150)	(0.126)
• *fdi*		−1.794	−5.351^***^		−0.357	−4.463^***^
		(1.824)	(1.525)		(1.798)	(1.511)
• ln*pop*		0.831^***^	0.828^***^		0.840^***^	0.847^***^
		(0.017)	(0.014)		(0.015)	(0.013)
• *gov*		−0.476^***^	0.141		−0.764^***^	0.017
		(0.108)	(0.090)		(0.113)	(0.095)
• ln*rgdp*		0.181^***^	0.212^***^		0.205^***^	0.223^***^
		(0.015)	(0.013)		(0.015)	(0.013)
• Constant	10.117^***^	0.301^**^	0.691^***^	0.767^***^	0.121	0.676^***^
	(0.046)	(0.123)	(0.103)	(0.005)	(0.128)	(0.108)
• City FE	Y	Y	Y	Y	Y	Y
• Year FE	Y	Y	Y	Y	Y	Y
• Observations	2,558	2,558	2,558	2,558	2,558	2,558
• *F*-value	90.733	1,903.247	2,704.147	116.481	1,917.399	2,695.549

Standard errors are reported in in parentheses.

***, **, and * denote p < 0.01, p < 0.05 and p < 0.1.

According to the estimation results in Table 3, the estimated coefficients of *ri* in columns (1) and (4) are 0.304 (*p* < 0.01) and 0.040 (*p* < 0.01), respectively, indicating that regional integration will significantly promote transportation infrastructure improvement and industrial upgrading. Meanwhile, the regression coefficients for ln*tran* in columns (2) and (3) are 0.029 (*p* < 0.01) and 0.023 (*p* < 0.01), indicating that regional integration enhances the healthcare environment through improving transportation infrastructure. The coefficients of *is* in columns (5) and (6) are 0.359 (*p* < 0.01) and 0.097 (*p* < 0.1), indicating that regional integration enhances the healthcare environment through industrial upgrading. The above results suggest that regional integration can enhance the healthcare environment through improving transportation infrastructure and promoting industrial upgrading. Therefore, government should implement more effective industrial policies and transportation planning in the regional integration process to expand the resulting improvements in the healthcare environment.

### 4.3. Differences in the impact of regional integration on core cities and peripheral cities

To test hypothesis 3, i.e., whether regional integration has a stronger effect on enhancing the public healthcare environment in core cities than in peripheral cities, the following HLM model with interaction term *ri* × *cp* is constructed. *cp*_i,t_ denotes a dummy variable for core cities and peripheral cities, and takes the value of 1 when city *i* is the core city, otherwise it takes the value of 0. The estimation results are reported in Table 4, where the dependent variables in columns (1) and (2) are healthcare workforce, and in columns (3) and (4) are healthcare infrastructure.

**Table 4:** Impact of regional integration on public healthcare environment in core cities and peripheral cities.

	**ln*phe1***		**ln*phe2***	
	**(1)**	**(2)**	**(3)**	**(4)**
• *ri*	0.361^***^	0.127^***^	0.379^***^	0.061^***^
	(0.019)	(0.011)	(0.020)	(0.010)
• *cp*	1.074^***^	0.349^***^	0.972^***^	0.278^***^
	(0.074)	(0.038)	(0.080)	(0.035)
• *ri* × *cp*	0.155^***^	0.095^***^	0.173^***^	0.083^***^
	(0.054)	(0.017)	(0.059)	(0.014)
• ln*ict*		0.077^***^		0.095^***^
		(0.010)		(0.009)
• *gie*		0.185		−0.496^***^
		(0.150)		(0.127)
• *fdi*		−12.752^***^		−8.474^***^
		(1.733)		(1.435)
• ln*pop*		0.815^***^		0.809^***^
		(0.014)		(0.012)
• *gov*		0.718^***^		0.396^***^
		(0.138)		(0.088)
• ln*rgdp*		0.322^***^		0.232^***^
		(0.019)		(0.012)
• Constant	8.113^***^	−0.179	8.882^***^	1.066^***^
	(0.053)	(0.183)	(0.029)	(0.098)
• ${\sigma_{\mu 0}}^{2}$	0.538^***^	0.244^***^	0.498^***^	0.210^***^
	(0.033)	(0.017)	(0.031)	(0.014)
• σ^2^	0.184^***^	0.176^***^	0.299^***^	0.138^***^
	(0.003)	(0.003)	(0.004)	(0.002)
• City FE	Y	Y	Y	Y
• Year FE	Y	Y	Y	Y
• Observations	2,558	2,558	2,558	2,558
• Likelihood ratio	4,820.02	1,504.56	2,856.50	2,222.45

Standard errors are reported in in parentheses.

*** denote p < 0.01.

$\sigma_{\mu 0}^{2}$ and σ^2^ denote intra-cluster variance and the inter-cluster variance. Likelihood ratio denotes the test comparing HLM and linear model.

According to the results in Table 4, the regression coefficient of the interaction term *ri* × *cp* in column (2) is 0.095, indicating that the effect of regional integration on the enhancement of medical workforce in core cities is 9.5% higher than that in peripheral cities. The regression coefficient of the interaction term *ri* × *cp* in column (4) is 0.083, indicating that the effect of regional integration on the improvement of medical infrastructure in core cities is 8.3% higher than that in peripheral cities. This implies that regional integration can significantly improve the overall public healthcare environment in the region, but its effect is higher in the core cities than in the peripheral cities.

### 4.4. Heterogeneity analysis

#### 4.4.1. Heterogeneity analysis of different levels of economic development

The level of economic development has a critical impact on the healthcare environment. In more economically developed areas, the government has higher financial expenditures to improve healthcare services. At the same time, economically developed areas have a stronger demand for healthcare, which will attract more healthcare providers to enter and thus improve the local healthcare environment (9). Therefore, in assessing the impact of regional integration on healthcare environment, it is necessary to consider its differences across regions with different levels of economic development. This paper divides the sample into high economic development level group and low economic development level group based on the mean value of GDP per capita for group regression. The regression results are reported in Table 5.

**Table 5:** Heterogeneity analysis of different levels of economic development.

	**High economic development level**		**Low economic development level**	
	**ln*phe1***	**ln*phe2***	**ln*phe1***	**ln*phe2***
	**(1)**	**(2)**	**(3)**	**(4)**
• *ri*	0.015	0.018^*^	0.076^**^	0.052^*^
	(0.011)	(0.010)	(0.037)	(0.029)
• ln*ict*	0.200^***^	0.175^***^	0.096^***^	0.112^***^
	(0.014)	(0.012)	(0.016)	(0.012)
• *gie*	−0.711^***^	−1.352^***^	−0.377^*^	−0.828^***^
	(0.204)	(0.184)	(0.226)	(0.176)
• *fdi*	−7.601^***^	−7.572^***^	−19.256^***^	−18.847^***^
	(2.102)	(1.898)	(3.298)	(2.570)
• ln*pop*	0.828^***^	0.857^***^	0.857^***^	0.810^***^
	(0.017)	(0.016)	(0.023)	(0.018)
• *gov*	0.765^***^	0.343^**^	2.073^***^	1.955^***^
	(0.173)	(0.156)	(0.283)	(0.220)
• ln*rgdp*	0.456^***^	0.286^***^	0.354^***^	0.387^***^
	(0.025)	(0.023)	(0.036)	(0.028)
• Constant	−3.156^***^	−0.418^*^	−0.744^**^	−0.320
	(0.263)	(0.237)	(0.343)	(0.267)
• ${\sigma_{\mu 0}}^{2}$	0.538^***^	0.244^***^	0.498^***^	0.210^***^
	(0.033)	(0.017)	(0.031)	(0.014)
• σ^2^	0.184^***^	0.176^***^	0.299^***^	0.138^***^
	(0.003)	(0.003)	(0.004)	(0.002)
• City FE	Y	Y	Y	Y
• Year FE	Y	Y	Y	Y
• Observations	1,552	1,552	1,006	1,006

Standard errors are reported in in parentheses.

***, **, and * denote p < 0.01, p < 0.05 and p < 0.1.

$\sigma_{\mu 0}^{2}$ and σ^2^ denote intra-cluster variance and the inter-cluster variance.

According to the regression results in Table 5, the regression coefficients of ***ri*** in columns (1) and (2) are 0.015 and 0.018, respectively, and only column (2) passes the 1% significance level test. And the regression coefficients of ***ri*** in columns (3) and (4) are 0.076 and 0.052, which pass the significance level test of 5 and 10%, respectively. The above results indicate that regional integration has a stronger effect on enhancing public healthcare environment in cities with lower levels of economic development.

#### 4.4.2. Heterogeneity analysis of different public healthcare environment levels

To further test whether there are differences in the effects of regional integration on regions with different levels of public healthcare environment, this paper divides the sample into two groups for group regression according to the high and low public healthcare environment. The grouping is based on the mean value of public healthcare environment for all cities in the year, with those above the mean value being included in the high-level group and those below the mean value being included in the low-level group. The estimated results are shown in Table 6.

**Table 6:** Heterogeneity analysis of different public healthcare environment levels.

	**High public healthcare environment**		**Low public healthcare environment**	
	**ln*phe1***	**ln*phe2***	**ln*phe1***	**ln*phe2***
	**(1)**	**(2)**	**(3)**	**(4)**
• *ri*	−0.005	−0.038^***^	0.143^***^	0.075^***^
	(0.013)	(0.010)	(0.017)	(0.015)
• ln*ict*	0.184^***^	0.176^***^	0.046^***^	0.089^***^
	(0.016)	(0.013)	(0.011)	(0.011)
• *gie*	−0.609^**^	−1.525^***^	−0.130	−0.419^**^
	(0.243)	(0.181)	(0.163)	(0.167)
• *fdi*	−16.461^***^	−10.464^***^	−6.378^***^	−11.274^***^
	(2.637)	(2.012)	(2.032)	(2.050)
• ln*pop*	0.694^***^	0.760^***^	0.717^***^	0.724^***^
	(0.022)	(0.018)	(0.018)	(0.018)
• *gov*	0.820^***^	0.167	1.052^***^	1.287^***^
	(0.209)	(0.162)	(0.164)	(0.164)
• ln*rgdp*	0.339^***^	0.268^***^	0.268^***^	0.257^***^
	(0.028)	(0.022)	(0.022)	(0.022)
• Constant	−0.278	0.706^***^	1.154^***^	1.464^***^
	(0.276)	(0.214)	(0.239)	(0.234)
• ${\sigma_{\mu 0}}^{2}$	0.538^***^	0.244^***^	0.498^***^	0.210^***^
	(0.033)	(0.017)	(0.031)	(0.014)
• σ^2^	0.184^***^	0.176^***^	0.299^***^	0.138^***^
	(0.003)	(0.003)	(0.004)	(0.002)
• City FE	Y	Y	Y	Y
• Year FE	Y	Y	Y	Y
• Observations	1,204	1,283	1,354	1,275

Standard errors are reported in in parentheses.

*** and ** denote p < 0.01 and p < 0.05.

$\sigma_{\mu 0}^{2}$ and σ^2^ denote intra-cluster variance and the inter-cluster variance. Likelihood ratio denotes the test comparing HLM and linear model.

According to the regression results in Table 6, the regression coefficients of *ri* in columns (1) and (2) are −0.005 and −0.038. The regression coefficient in column (1) does not pass the 10% significance level test, and the regression coefficient in column (2) passes the 1% significance level test. The regression coefficients of *ri* in columns (3) and (4) are 0.143 and 0.075, both of which pass the 1% significance level test. The above results indicate that regional integration has a stronger effect on the enhancement of areas with lower levels of public healthcare environment. This implies that the promotion of regional integration not only enhances the regional public healthcare environment but also reduces the gap of public healthcare environment within the region.

### 4.5. Endogeneity test

**Figure 1:**
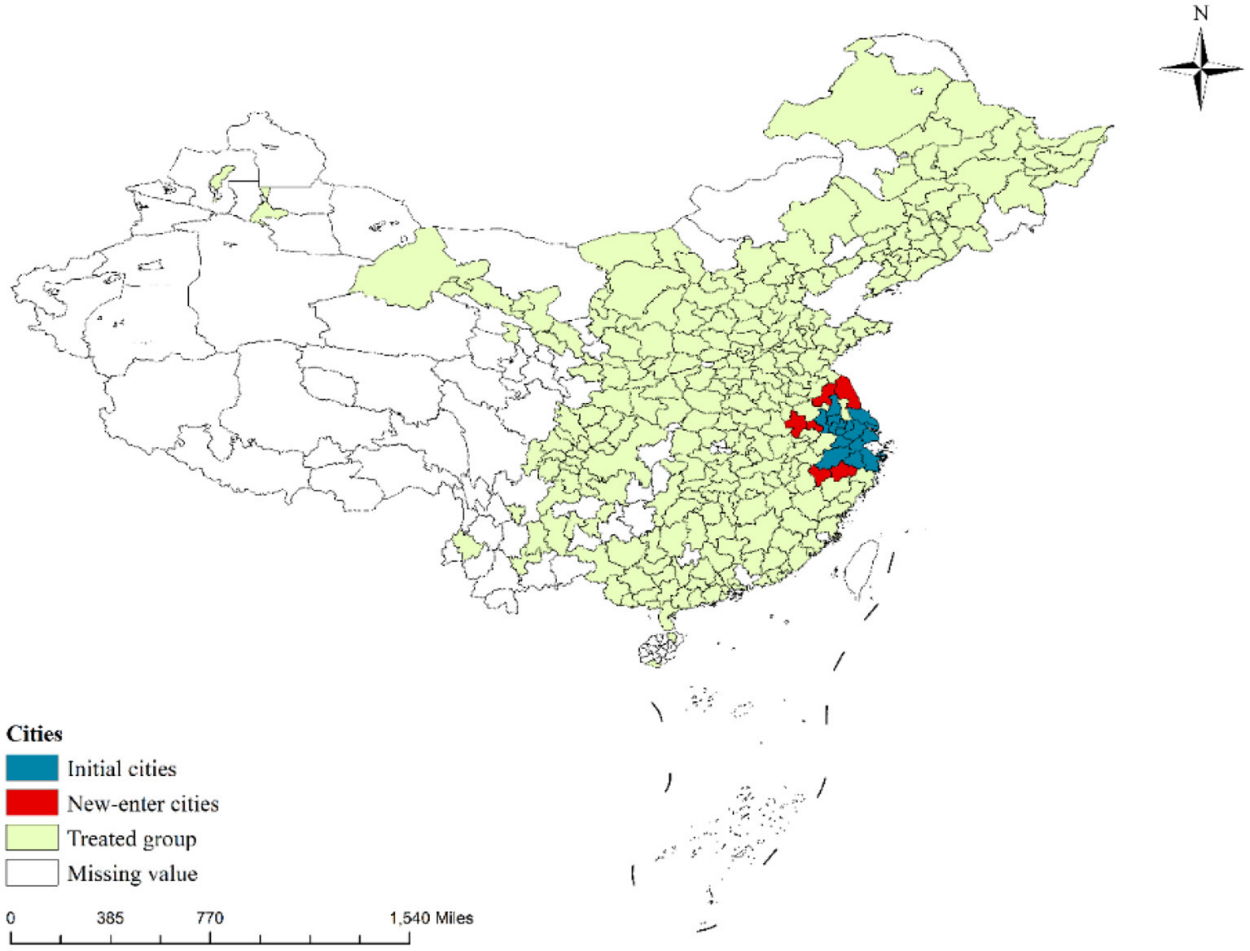
Spatial distribution of Yangtze River Delta expansion.

In this paper, the functional specialization division of labor index is selected to measure regional integration. This index and public healthcare environment are influenced by the level of economic development, which may lead to endogenous interference and reduce the reliability of the results. Therefore, this paper selects Yangtze River Delta Expansion (YRDE) as a quasi-natural experiment to develop an endogeneity test by difference-in-difference (DID) model estimation. The Yangtze River Delta city cluster is one of the most representative cities in China. With the strengthening and deepening of economic ties between cities in and outside the Yangtze River Delta region, the Yangtze River Delta has steadily expanded into northern Jiangsu, southern Zhejiang, and eastern Anhui. In March 2010, the tenth meeting of the Yangtze River Delta Economic Coordination Council (YRDECC) officially absorbed six cities, namely, Hefei, Yancheng, Maanshan, Jinhua, Huaian, and Quzhou, as members (see Figure 1). This paper will use this expansion as a quasi-natural experiment to assess the impact of regional integration on public healthcare environment to ensure the robustness of the results in this paper.

Table 7 below reports the results of the endogeneity test, where YRDE is a policy shock variable that takes the value of 1 when city *i* is implemented in year *t* and 0 otherwise. According to the estimation results in Table 6, the coefficients of the impact of *YRDE* on healthcare workforce and healthcare infrastructure were 0.127 (*p* < 0.01) and 0.057 (*p* < 0.01), respectively. This suggests that the implementation of the YRDE policy significantly enhances public healthcare environment. Therefore, the results of the analysis in this paper are still reliable after excluding potential endogenous disturbances.

**Table 7:** Endogeneity test based on DID model.

	**ln*phe1***		**ln*phe2***	
	**(1)**	**(2)**	**(3)**	**(4)**
• *YRDE*	1.056^***^	0.127^***^	0.951^***^	0.057^***^
	(0.045)	(0.021)	(0.043)	(0.017)
• ln*ict*		0.136^***^		0.140^***^
		(0.011)		(0.009)
• *gie*		−0.343^**^		−1.138^***^
		(0.155)		(0.129)
• *fdi*		−14.424^***^		−12.218^***^
		(1.848)		(1.541)
• ln*pop*		0.853^***^		0.846^***^
		(0.014)		(0.012)
• *gov*		0.866^***^		0.670^***^
		(0.145)		(0.121)
• ln*rgdp*		0.384^***^		0.293^***^
		(0.019)		(0.016)
• Constant	8.688^***^	−1.325^***^	9.200^***^	0.189
	(0.061)	(0.186)	(0.058)	(0.155)
• ${\sigma_{\mu 0}}^{2}$	0.710^***^	0.341^***^	0.693^***^	0.306^***^
	(0.043)	(0.025)	(0.042)	(0.022)
• σ^2^	0.184^***^	0.177^***^	0.126^***^	0.117^***^
	(0.003)	(0.003)	(0.002)	(0.002)
• City FE	Y	Y	Y	Y
• Year FE	Y	Y	Y	Y
• Observations	2,558	2,558	2,558	2,558

Standard errors are reported in in parentheses.

*** denote p < 0.01.

$\sigma_{\mu 0}^{2}$ and σ^2^ denote intra-cluster variance and the inter-cluster variance.

**Figure 2:**
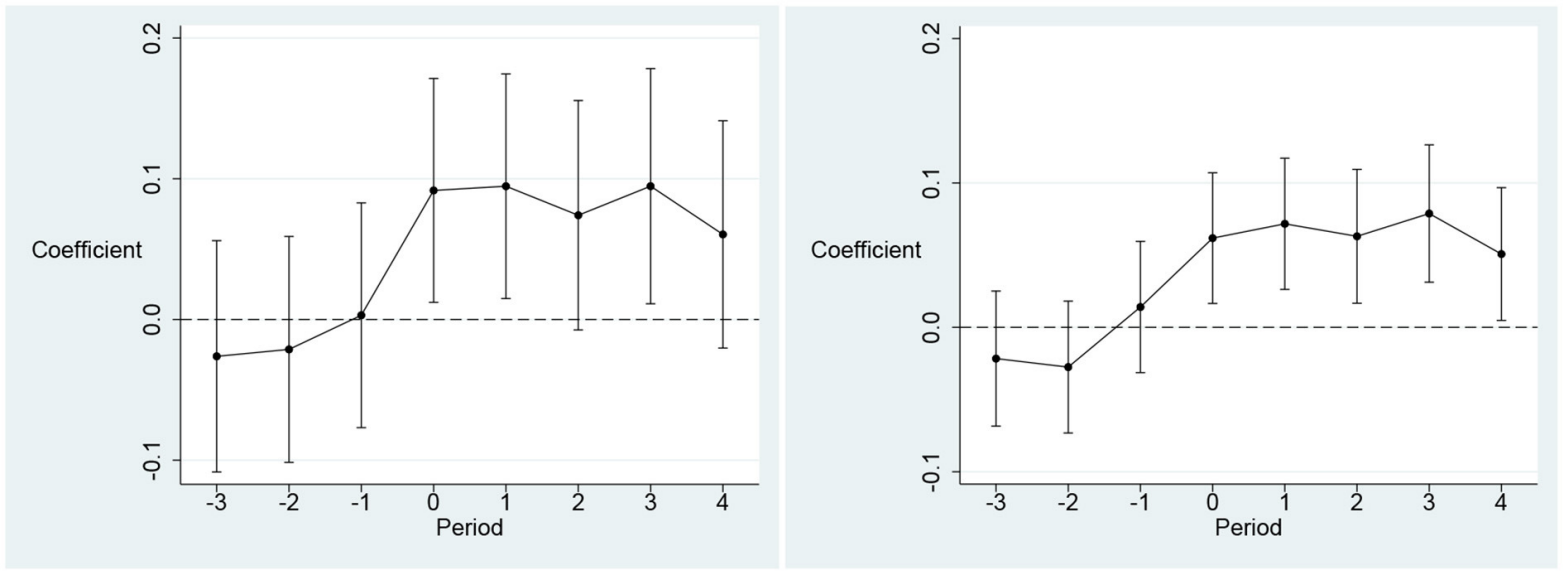
Results of Parallel Trend Test. The dependent variable in the left panel is healthcare workforce (ln*phe*1) and the dependent variable in the right panel is healthcare infrastructure (ln*phe*2). The X-axis denotes the window period for YRDE implementation. The Y axis denotes the coefficient of YRDE. The year before YRDE is implemented as the base period.

Table 7 reports the results of the endogeneity test based on the DID model. The results hold provided that the parallel trend hypothesis test is satisfied, i.e., there is a common trend in the healthcare environment between the treatment and control groups before and after *YRDE* implementation. The results of the parallel trend test are reported in Figure 2. Before the implementation of *YRDE*, the regression coefficients mostly did not pass the significance test at the 5% level. This indicates that there was no significant difference in the public healthcare environment between the control group and treatment group before the implementation of BCCP. This indicates that the parallel trend hypothesis was met, i.e., the endogeneity test based on the DID model supported the findings in Section 4.1.

### 4.6. Robustness test

To ensure the robustness of the results, this paper also uses a high-dimensional fixed-effects model for re-estimation. We consider both city cluster fixed effects, city fixed effects and year fixed effects in the model, and the results are shown in Table 8.

**Table 8:** Re-estimation based on high-dimensional fixed-effects model.

	**ln*phe1***		**ln*phe2***	
	**(1)**	**(2)**	**(3)**	**(4)**
• *ri*	0.066^***^	0.055^***^	0.345^***^	0.067^***^
	(0.020)	(0.017)	(0.033)	(0.016)
• ln*ict*		0.001		0.046^***^
		(0.012)		(0.013)
• *gie*		0.236		1.123^***^
		(0.319)		(0.249)
• *fdi*		−6.187^**^		−5.509^*^
		(2.849)		(2.827)
• ln*pop*		0.633^***^		0.619^***^
		(0.139)		(0.127)
• *gov*		−0.553^*^		1.339^***^
		(0.297)		(0.255)
• ln*rgdp*		0.017		0.264^***^
		(0.053)		(0.021)
• Constant	8.847^***^	4.939^***^	9.128^***^	2.110^***^
	(0.026)	(1.195)	(0.042)	(0.682)
• City FE	Y	Y	Y	Y
• City cluster FE	Y	Y	Y	Y
• Year FE	Y	Y	Y	Y
• Observations	2,558	2,558	2,558	2,558
• F-value	10.763	9.511	111.655	254.328

***, **, and * denote *p* < 0.01, *p* < 0.05, and *p* < 0.1.

According to the estimation results in Table 7, the regression coefficients of *ri* in columns (2) and (4) are 0.055 (*p* < 0.01) and 0.067 (*p* < 0.01). This indicates that the increase in regional integration significantly enhances public healthcare environment, i.e., the results of this paper are robust.

## 5. Conclusions and recommendations

Based on panel data for 137 cities in 16 major urban agglomerations from 2001 to 2019, this paper assesses the impact of regional integration on the public healthcare environment using a hierarchical linear model (HLM). Our findings indicate that a 1% increase in regional integration leads to an average increase of 6.6 and 1.9% in urban healthcare workforce and healthcare infrastructure. In addition, regional integration will also enhance the positive impact of population and economic development on public healthcare environment. Our findings also suggest that regional integration has a higher effect on enhancing the public healthcare environment in core cities than in peripheral cities. The results of the mechanism analysis indicate that regional integration affects the public healthcare environment through improving transportation infrastructure and industrial upgrading. Further heterogeneity analysis shows that regional integration has stronger positive effect on public healthcare environment in cities with lower level of economic development and public healthcare environment. Finally, the results of the endogeneity test and the robustness test yielded a more consistent conclusion.

Based on the findings of this paper, we can propose three recommendations. First, the government should develop a more comprehensive regional cooperation plan to improve the public healthcare environment. The public healthcare environment is a basic guarantee for improving public health, and the government needs to improve the public healthcare environment both directly through financial expenditures and by guiding market participation to improve the public healthcare environment. The findings of this paper suggest that regional integration can bring positive impact to enhance the public healthcare environment. Therefore, the government should introduce more regional cooperation programs to promote regional integration, such as the plan of Guangdong-Hong Kong-Macao Greater Bay Area and Yangtze River Delta Expansion.^2^ Second, financial spending on improving the healthcare environment in peripheral cities should be increased. Our findings suggest that regional integration has a significantly higher effect on improving public healthcare environment in core cities than in peripheral cities. This can lead to a widening of the gap in the regional medical public health environment. Therefore, the government should strengthen fiscal spending on peripheral cities to reduce the regional public healthcare environment gap. Specifically, the government can implement living subsidies and social security for talents imported from hospitals to enhance the public healthcare environment in peripheral cities. Finally, regional integration policy development needs to consider differences across regions. Our findings show that regional integration has a stronger impact on regions with lower levels of economic development and public healthcare environment. Therefore, the government should focus on regional integration planning in developing regions, such as the Guanzhong-Tianshui city cluster. Promoting integration in such regions can enhance the local public healthcare environment more effectively.

## Data availability statement

The raw data supporting the conclusions of this article will be made available by the authors, without undue reservation.

## Author contributions

CT: conceptualization, methodology, and writing–original draft. YZe: writing–review and editing and data analysis. HL: writing–original draft, data analysis, and software. CY: data curation, funding acquisition, supervision, validation, project administration, and writing–original draft. YT, YZh, and CZ: writing–review and editing. All authors contributed to the article and approved the submitted version.

## Footnotes

^1^The Yangtze River Delta Expansion refers to a quasi-natural policy, the main element of which is the expansion of the geographical area of the Yangtze River Delta urban agglomeration. This paper shows in detail in the appendix the geographical location of the Yangtze River Delta city cluster and the cities included. In March 2010, the tenth meeting of the Yangtze River Delta Economic Coordination Council officially absorbed six cities, namely, Hefei, Yancheng, Maanshan, Jinhua, Huaian, and Quzhou, as members. This paper elaborates on the policy in Section 4.4 and shows the scope of the policy implementation with a map.

^2^Guangdong-Hong Kong-Macao Greater Bay Area and Yangtze River Delta are the two major urban agglomerations in China's coastal region and the two most economically developed urban agglomerations in China. This paper shows in the appendix the geographical location of the above two urban agglomerations and the cities they contain.
