# Supplementary material for: Regional integration and public healthcare environment: Evidence from China
Source: Front Public Health. 2023 Jan 4;10:1013053. doi: 10.3389/fpubh.2022.1013053 (PMC9846215; doi:10.3389/fpubh.2022.1013053)
Supplement: Supplementary file 1 [file Table_1.docx]

**Table A1**

Cities contained in city clusters

| City clusters | Cities | City clusters | Cities |
| --- | --- | --- | --- |
| BTHCC | Tangshan, Zhangjiakou, Chengde, **Tianjin**, Qinhuangdao  Shijiazhuang, Baoding, Cangzhou, **Beijing** and Langfang | CPCC | Jiaozuo, Luoyang, **Zhengzhou**, Pingdingshan, Xinxiang, Kaifeng, Xuchang and Luohe |
| YRDCC | Jiaxing, Ningbo, Changzhou, **Shanghai**, Suzhou, Wuxi, Hangzhou, Zhenjiang, Shaoxing, Nanjing, Huzhou, Nantong, Taizhou, Zhoushan and Yangzhou | WCC | Huangshi, Ezhou, **Wuhan**, Xianning, Huanggang and Xiaogan |
| PRDCC | Dongguan, Foshan, **Shenzhen**, Zhongshan, Huizhou, Jiangmen, Zhuhai, **Guangzhou** and Zhaoqing | CZTCC | Loudi, Xiangtan, Hengyang, Zhuzhou, Yueyang, Yiyang, **Changsha** and Changde |
| SCLCC | Benxi, Anshan, Liaoyang, Yingkou, Fushun, Dalian, Panjin, Dandong, **Shenyang** and Tieling | PLCC | Xinyu, Jiujiang, **Nanchang**, Ji'an, Jingdezhen, Fuzhou, Yichun, Shangrao and Yingtan |
| SPCC | Zibo, Dongying, Rizhao, Weifang, **Jinan**, Yantai, Qingdao and Weihai | CCCC | Leshan, Meishan, **Chongqing**, Yibin, Dazhou, Deyang, Ya'an, Luzhou, Zigong, **Chengdu**, Mianyang, Nanchong, Suining, Ziyang, Guang'an and Neijiang |
| HCCC | Daqing, Jilin, Mudanjiang, Qiqihar, **Harbin**, Songyuan and **Changchun** | GTCC | Tongchuan, Baoji, **Xi'an**, Xianyang, Weinan, Shangluo and Tianshui |
| JCC | Ma'anshan, Tongling, Anqing, Chizhou, Wuhu, Chaohu, Chuzhou, Xuancheng and **Hefei** | TCC | **Taiyuan**, Jinzhong, Xinzhou and Luliang |
| WSSCC | Sanming, Nanping, Zhangzhou, Longyan, **Xiamen**, Quanzhou, Fuzhou, Putian and Ningde | BGCC | Fangchenggang, Qinzhou, **Nanning** and Beihai |

Note: Cities with black bold are core cities, and the cities without black bold are the peripheral cities
