# Supplementary figures and images for: Regional integration and public healthcare environment: Evidence from China
Source: Front Public Health. 2023 Jan 4;10:1013053. doi: 10.3389/fpubh.2022.1013053 (PMC9846215; doi:10.3389/fpubh.2022.1013053)

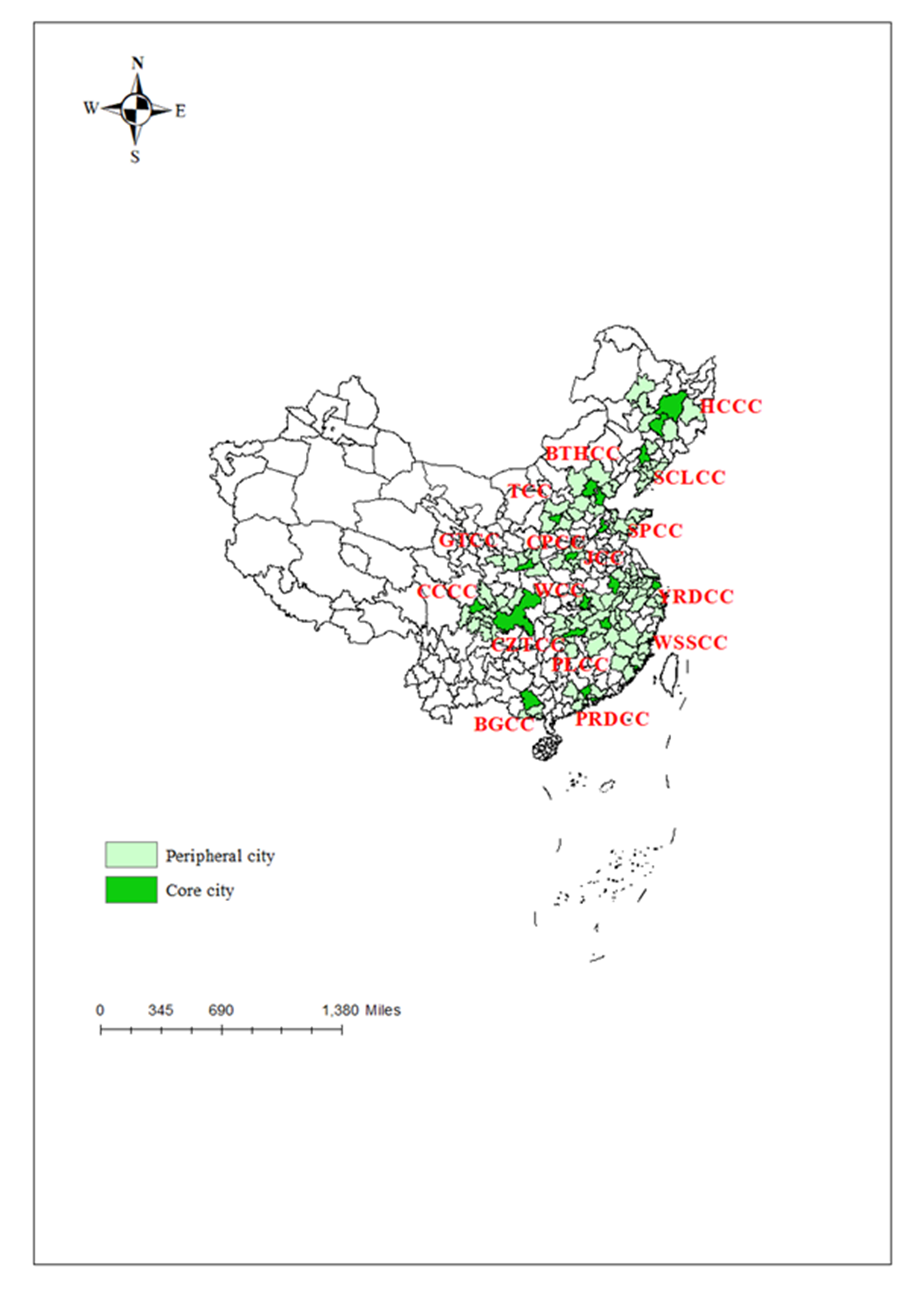

Supplement: Supplementary file 2 [file Image_1.tif]
